# Heavy commercial vehicles’ disposition: Anonymized dataset of German truck freight transport order trips (DT-DISPO)

**DOI:** 10.1016/j.dib.2026.113019

**Published:** 2026-06-23

**Authors:** Tom Winkler, Marcel Brödel, Laura Heindl, Niclas Klein, Anna Paper

**Affiliations:** Technical University of Munich, TUM School of Engineering and Design, Chair of Automotive Technology, Boltzmannstr. 15, 85748 Garching, Germany

**Keywords:** Road freight transport, Heavy-duty vehicle, Fleet data, Logistics, Dispatching, Disposition

## Abstract

This article presents an anonymized dataset of real-world transport order trip data spanning a total operational period of 16 months. The original data has been provided by two German freight forwarding companies as an export from their respective Transport Management System (TMS). All personal and company-identifiable information has been anonymized to ensure privacy while maintaining relational integrity for research purposes. The dataset comprises 31,352 disposition records from two fleet operators: one with a fleet of 37 heavy-duty vehicles spanning a continuous 5-month period in 2024, and the second with 83 heavy-duty vehicles spanning a continuous 10-month period in 2024. Both fleets comprise only internal-combustion engine vehicles. The dataset provides granular data regarding start and end locations (coordinates), schedule time windows, driving distances, allocated vehicles, and allocated drivers. This dataset can be used by researchers in logistics and transportation engineering to develop and benchmark vehicle routing and disposition/dispatching algorithms, analyse freight transport behaviour, develop demand prediction models, or novel electric vehicle disposition modelling.

Specifications Table

**Table:** 

Subject	Engineering & Materials science
Specific subject area	Road freight transport logistics management
Type of data	Table (.csv format)
Data collection	The original data were exported from the transport management systems (TMS) of two distinct German freight forwarding companies. This data was filtered to remove records with missing data for start and end location and schedule time window. The filtered data was anonymized by replacing the original values for the schedule time window date, the allocated vehicle, and the allocated driver with pseudo-anonymized values. The data for the start and end locations were anonymized using two spatial approaches of the clustered-isometry-method and the transient-shift-method.
Data source location	Country: Germany
Data accessibility	Repository name: TUMFTM/heavy-duty-vehicle-order-dataset Data identification number: 10.5281/zenodo.19052657 Direct URL to data: https://zenodo.org/records/19052657 Instructions for accessing these data: The repository contains one csv files. A concise description of its content is provided in Chapter “Data Description” within this manuscript.
Related research article	none

## 1 Value of the Data

• The dataset provides real-world structured records that reflect how heavy commercial road vehicle freight orders are represented for disposition/dispatching, enabling method development on realistic field formats and constraints.
• The dataset includes location, time, distance, driver, and vehicle information that can be reused to benchmark disposition/dispatching-, routing-, scheduling-, and resource-assignment-algorithms.
• The dataset enables studies of depot interactions, delivery geography, and operational patterns via consistent date/time and location information.
• The geographic data allows for the analysis of freight truck flow patterns, enabling the identification of spatial patterns and trip-to-route assessments.
• The temporal data can be used to train machine learning models for predicting freight truck flow peaks, daily time-of-day patterns, and day-of-week variations.

## 2 Background

The dataset stems from research into heavy commercial and electrified vehicle disposition algorithms at the Technical University of Munich Chair of Automotive Technology. Disposition / dispatching of heavy-duty road freight typically requires converting transport demand into vehicle- and tour-level plans under time, capacity, and geographic constraints. The dataset provided has been compiled using real-world operational data from logistics partners to address the lack of publicly available freight transport records. The fleets consist solely of heavy-duty commercial vehicles in EU category “N3” (corresponding to US “Class 8”) with gross vehicle weight ratings exceeding 12 tons. It can be used for the development and validation of such disposition algorithms, but also to support other research areas within the logistics and transportation engineering.

## 3 Data Description

The dataset is provided as a single comma separated value (CSV) file, containing a total of 31,352 individual trips, distributed across 2 freight forwarders, 285 unique days, and 120 unique trucks. The data is provided online [1].

The main dataset file “dataset_hdv_order_data.csv” contains records related to specific real-world trips with each trip parameters described in Table 1.

**Table 1:** Dataset column and trip parameter description.

Dataset column	Unit	Data type	Description
forwarder_id	-	int	unique sequential identifier for the original freight forwarder, 1 or 2
day_id	-	int	unique sequential identifier of operational day, 1 to 285
trip_id	-	int	unique sequential identifier for a single daily trip within each day
truck_id	-	int	unique sequential vehicle identifier for the original truck license plate, 1 to 84, N/A possible where data not available
driver_id	-	int	unique sequential identifier for the assigned driver, 1 to 32, N/A possible where data not available
trip_window_start_time_hhmm	HH:MM	str	start of trip time window (earliest pick-up time), 00:00 to 23:59
trip_window_end_time_hhmm	HH:MM	str	end of trip time window (latest drop-off time), 00:00 to 23:59
trip_start_coords_lon	decimal degree	dec	trip start longitude coordinate (pick-up location), WGS84 reference system format
trip_start_coords_lat	decimal degree	dec	trip start latitudinal coordinate (pick-up location), WGS84 reference system format
trip_end_coords_lon	decimal degree	dec	trip end longitude coordinate (drop-off location), WGS84 reference system format
trip_end_coords_lat	decimal degrees	dec	trip end latitudinal coordinate (drop-off location), WGS84 reference system format
duration_day_count_d	days	int	trip event duration in full days, 1 to 8
trip_distance_km	km	int	routed trip distance
trip_duration_hhmm	HH:MM	str	routed trip duration

**Fig. 1:**
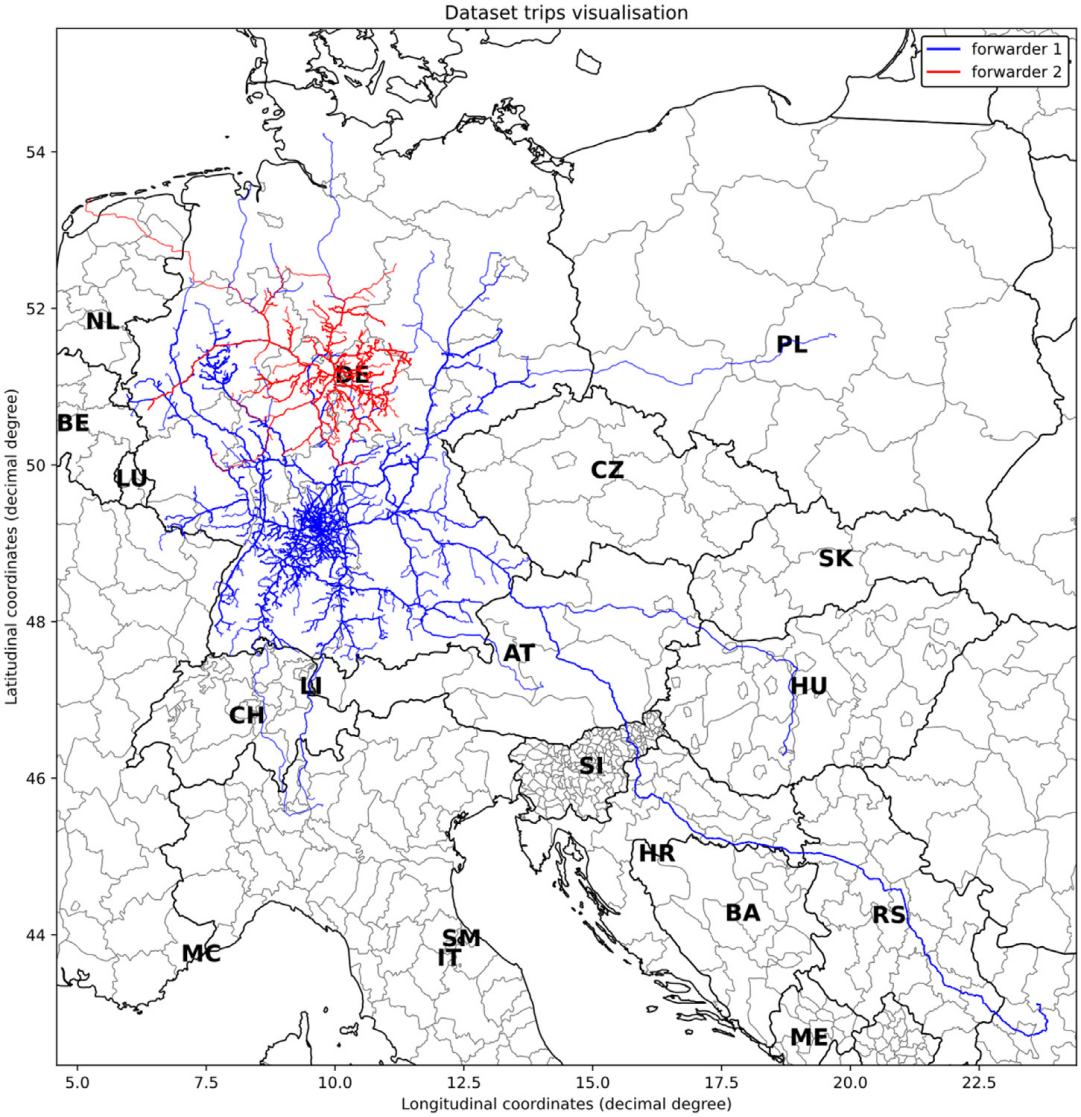
Anonymized routed trips across Europe for freight forwarder 1 (blue) and freight forwarder 2 (red).

The visualized total trip set is shown in Fig. 1 on an overview map with the routed trips for both freight forwarders.

## 4 Experimental Design, Materials and Methods

### 4.1 Data acquisition

The original data were exported from the transport management systems (TMS) of two distinct German freight forwarding companies (FF) operating in the heavy-duty vehicle sector. FF 1 provided disposition records spanning a continuous 5-month operational period in 2024, while FF 2 contributed data covering a continuous 10-month period in 2024. Both FFs operate exclusively internal combustion engine vehicle fleets within Germany.

The raw datasets comprised structured tabular records containing trip-level information including vehicle identifiers, driver assignments, temporal windows, geographic origin-destination pairs, and route characteristics. FF 1′s dataset initially contained 10,670 records across 39 vehicles, while FF 2′s dataset included 30,329 records using 83 vehicles.

### 4.2 Data preprocessing, filtering, standardization, and harmonization

Prior to anonymization, the datasets underwent systematic cleaning to ensure data quality and relevance for transportation research applications. The preprocessing workflow, implemented in Python 3.13.1 using the pandas library (version 3.0.1), consisted of the following steps:

1. Records with null or invalid entries in critical fields, being trip start and end locations (postal code and city name) and time windows, were identified and excluded from further processing. This filtering ensured completeness of relational attributes necessary for disposition analysis.
2. Trips with distances below 5 km were systematically removed, as these predominantly represented yard movements, depot-internal logistics operations, or data artifacts. For FF 1, the original distance values from the TMS export served as the filtering criterion, as these proved more accurate for very short distances than subsequently computed routing distances. This procedure eliminated 1120 records (10.5%) from FF 1′s dataset and 49 records (0.16%) from FF 2′s dataset, all of which represented zero-distance entries.
3. After filtering, FF 1′s dataset comprised 9550 valid trip records from 37 unique vehicles and 32 unique drivers. FF 2′s dataset retained 30,280 records spanning 83 vehicles.

To enable consistent analysis across both freight forwarders despite structural differences in their TMS exports, the datasets were harmonized into a unified schema. The standardization process addressed the following transformations:

1. FF 1 provided explicit tour start and tour end window timestamps with associated dates, enabling identification of multi-day operations. FF 2 provided only delivery deadline timestamps. To reconcile these differences, standardized columns “trip_window_start_time_hhmm” and “trip_window_end_time_hhmm” were created. For FF 1, these directly mapped to the original tour time windows. For FF 2, the start time was set uniformly to 00:00 (reflecting implicit depot departure flexibility), while the end time captured the delivery deadline. A supplementary column “duration_day_count_d” encoded trip duration in days (1 for single-day trips, 2–8 for multi-day operations in FF 1; uniformly 1 for FF 2).
2. Each operational day was assigned a sequential day_id starting from 1, and trips within each day received sequential trip_id values. This dual-key system provides unique trip identification while preserving temporal ordering.
3. Origin and destination locations, originally specified as city names with postal codes, were geocoded to geographic coordinates (longitude, latitude) in the WGS 84 coordinate reference system (EPSG:4326) [2]. Geocoding was performed using the “Nominatim” geocoder (OpenStreetMap-based) via the Python geopy library (version 2.4.1). The geocoding process incorporated multiple address format variants to maximize match rates, and a cache mechanism prevented redundant API calls for recurring locations. For 18 locations that failed automated geocoding after multiple attempts, coordinates were manually verified and hard-coded. FF 1′s trips featured distinct origin (sender address) and destination (receiver address) coordinates, reflecting direct freight haul operations. FF 2′s trips uniformly originated from the company depot, reflecting a hub-and-spoke distribution model.
4. To ensure consistency across datasets and compatibility with anonymization procedures, all trip distances were recomputed via the Geoapify Routing Application-Programming-Interface (API) using the “heavy_truck” routing profile, which accounts for vehicle restrictions, road network suitability, and realistic routing for heavy goods vehicles. The routing API was queried with origin and destination coordinates, returning routed distances (in kilometers, rounded to integers) and estimated durations (HH:MM format). A multi-tier fallback hierarchy was implemented: if “heavy_truck” routing failed, the system attempted truck mode, followed by drive mode, and ultimately haversine distance as a last resort. Routing results were cached to minimize API calls and ensure deterministic reproducibility. For FF 1, the recomputed distances replaced the TMS-provided values to maintain methodological consistency.

### 4.3 Anonymization methodology

To enable public dataset release while protecting commercially sensitive and privacy-relevant information, a comprehensive anonymization framework was developed and applied to both datasets. The framework addressed temporal, categorical, and spatial attributes. First, the pseudonymization of categorical identifiers:

1. Original vehicle license plate numbers were replaced with sequential pseudonymized identifiers with an integer assigned in order of first appearance in the dataset. This mapping was applied consistently within each dataset, preserving vehicle-specific trip patterns.
2. Similarly, driver codes were replaced with sequential pseudonymized identifiers. For FF 2, where driver information was unavailable in the source data, the driver identifier field was populated with empty values to indicate explicit absence.
3. Each freight forwarding company was assigned a sequential integer identifier “forwarder_id”, removing company names and commercial branding.

Secondly, the temporal pseudonymization:

1. To prevent calendar-based re-identification while preserving temporal patterns critical for disposition analysis, absolute dates were removed and replaced with relative day indices. The “day_id” column provides a sequential counter of the trip-start operational day, starting from 1 and incrementing for each new calendar date in the dataset. Non-operational days, in both FF cases weekend days are omitted from the sequence, preventing inference of actual calendar dates. This transformation preserves day-to-day operational continuity, weekly patterns (via modulo-5 operations), and multi-day trip characteristics, while obscuring the specific year and season of data collection. For example, the day sequence “1, 2, 3, 4, 5, 6, 7, 8, 9, 10” represents two weeks with 5 working days and no Saturdays and Sundays. Each public holiday included at least one trip in the original dataset and was therefore not accounted for in the day sequence.
2. Time-of-day information of the trip windows start “trip_window_start_time_hhmm” and end “trip_window_end_time_hhmm” was retained in HH:MM format, as these reflect operational scheduling patterns essential for electric vehicle feasibility studies and do not uniquely identify temporal context when decoupled from calendar dates.

As the dataset only describes freight trips, not other services/maintenance/etc. trips, the start/stop locations are either a pickup, a delivery, or a central depot. More specifically, an alternating pickup/drop-off location for FF 1 and depot vs. customer sites for FF 2. These geographic coordinates constituted the most re-identification-sensitive attribute, as precise location pairs could be cross-referenced with business directories, facility databases, or prior knowledge of freight corridors. Two distinct spatial anonymization methods were developed and applied to the respective datasets of the two FFs, balancing privacy protection (displacement magnitude) and analytical utility (preservation of distance and directional patterns). For FF 1′s dataset, characterized by diverse origin-destination pairs reflecting direct freight haul operations, the Clustered Isometry with Pairwise Routing [3] method was selected after systematic evaluation of eight candidate approaches:

1. Unique geographic points (204 distinct start locations, 548 distinct end locations) were spatially clustered using the DBSCAN algorithm (Density-Based Spatial Clustering of Applications with Noise [4], scikit-learn implementation) with parameters eps = 15,000 m and min_samples = 2. DBSCAN was applied in the EPSG:25,832 (UTM Zone 32 N) projection to ensure metric distance calculations. This produced spatially coherent clusters of nearby locations, while isolated points formed singleton clusters. To prevent excessive cluster sizes that would reduce anonymization strength, clusters exceeding 20 points were recursively subdivided.
2. Each cluster underwent a randomized rigid-body transformation (isometry) comprising: Translation by a random displacement vector with magnitude uniformly sampled from 5000 to 10,000 m Rotation by a random angle uniformly sampled from −5° to +5° The transformation parameters were generated deterministically, ensuring reproducibility while preventing reverse-engineering. Importantly, both start and end locations within the same cluster were transformed identically, preserving intra-cluster spatial relationships and relative distances.
3. To mitigate distance distortion introduced by cluster transformations, masked endpoints were further refined using an iterative search procedure: For each unique trip route (origin-destination pair), the initial masked endpoint candidate was obtained from cluster transformation
4. The candidate was evaluated by routing from the masked start to the masked end using the Geoapify Routing API (heavy_truck mode). If the routed distance deviated from the original trip distance by more than a tolerance threshold (defined as $\text{max}(0.2\;\text{km},0.05\times d_{\text{orig}})$), the endpoint was adjusted iteratively. Adjustment employed radial search in directional wedges: candidate endpoints were generated at varying radii and angles (±22.5°, ±15°, ±8°, 0° from the cluster-transformed position) and re-routed. The candidate minimizing absolute distance error was selected, with radius scaling applied via bisection over up to 8 iterations. This routing-based refinement ensured that masked trip distances closely approximated original distances (median absolute error: 6.1 km, 90th percentile: 22.9 km), critical for realistic vehicle routing problem formulations and range analyses.

FF 2's operational structure, where all trips originated from a single depot, required a specialized developed anonymization approach that preserved the hub-and-spoke topology while obscuring the depot location and individual destinations:

1. All geographic points (depot and all delivery endpoints) were first subjected to a uniform coordinate shift. This global shift displaced the entire operational region, preventing geographic contextualization while preserving all relative spatial relationships.
2. Each unique delivery endpoint was anonymized using a candidate search procedure designed to maintain trip distance fidelity: A base radius $r_{0}$ was computed as $r_{0}=\text{max}\left(\frac{d_{\text{orig}}}{1.5},2000\;m\right)$, scaling with trip distance. Candidate masked endpoints were generated at radius $r_{0}$ in multiple directions. A base direction (vector from masked depot to globally-shifted original endpoint) plus angular perturbations of −22.5°, −15°, −8°, 0°, +8°, +15°, +22.5° was used. Each candidate was routed from the masked depot using the Geoapify Routing API. The candidate yielding routed distance closest to the original trip distance was selected. If no candidate satisfied the tolerance ($\text{max}(0.2\;\text{km},0.05\times d_{\text{orig}})$), the radius was adjusted via bisection search over multiple iterations.
3. As with Company 1, all endpoint transformations were cached by unique endpoint key, ensuring deterministic and consistent anonymization across repeated trips to the same destination.

**Fig. 2:**
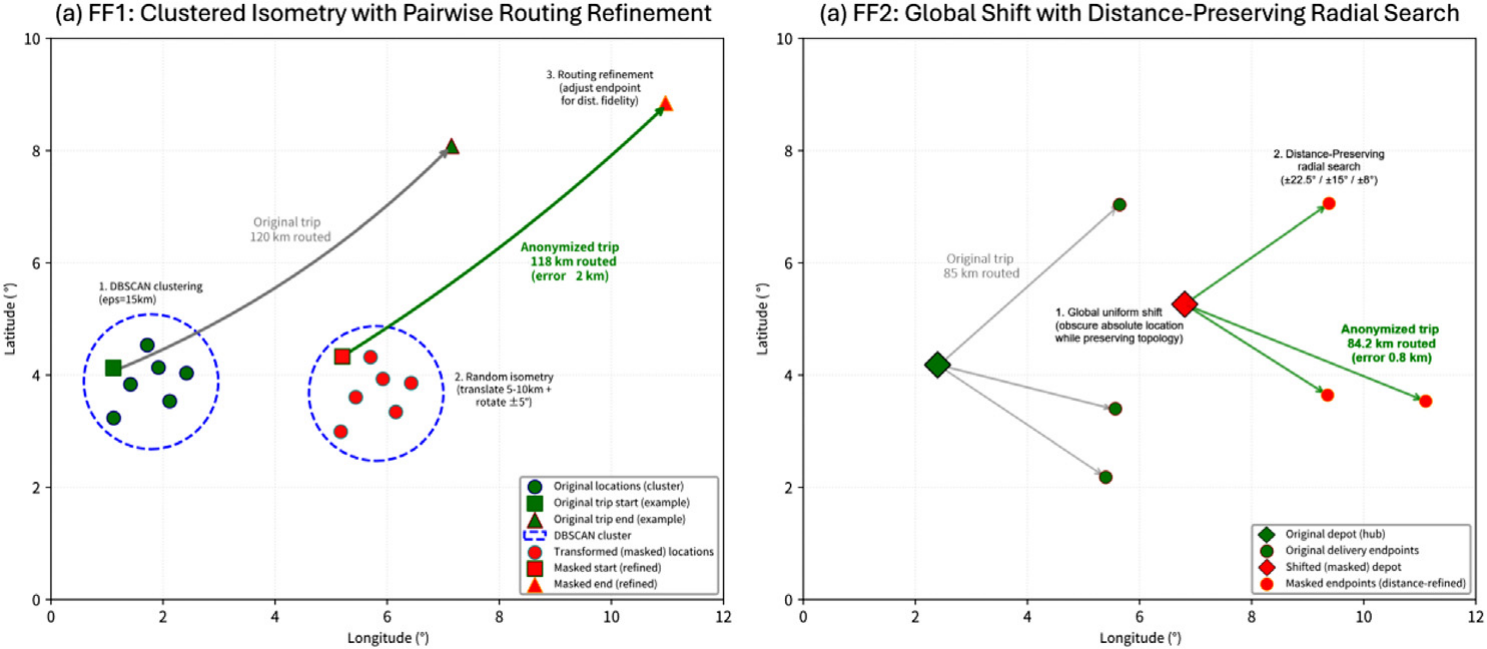
Schematic illustration of the two anonymization methods using a fictitious small-scale example. (a) Clustered Isometry with routing refinement representing the method applied to the data of freight forwarder 1 with DBSCAN clusters undergo rigid transformation (translation + small rotation) and endpoints locally adjusted to restore distance fidelity. (b) Global shift with per-endpoint radial search representing the method applied to the data of freight forwarder 2 with a uniform translation of the entire hub-and-spoke topology with angle/radius-adjustment of individual spokes from the new depot to preserve original trip distances within tolerance.

Both spatial anonymization methods are illustrated in Fig. 2 by using a fictional small-scale example to preserve the anonymity of the original data points.

### 4.4 Quantitative assessment of anonymization effects on data fidelity and research usability

The implemented anonymization methodology can result in deviations of key characteristics of the original dataset. Therefore, we performed systematic before-and-after comparisons on the key attributes most relevant to downstream transportation, logistics, and machine-learning analyses. All comparisons used the original (pre-anonymization) and final (post-anonymization) dataset. Because the anonymization procedures are deterministic and fully documented, these fidelity metrics are reproducible.

A two-sample Kolmogorov-Smirnov test was performed to analyze the distribution of routed trip distances before and after anonymization. [5] For FF 1, the trip distance distribution closely matches the original with a Kolmogorov–Smirnov statistic of 0.034 and a p-value of 0.38. With a Kolmogorov–Smirnov statistic of 0.028 and a p-value of 0.41, no significant difference was found for FF 2 either.

The median trip distance changed by < 4% for FF 1. The median absolute error (MAE) between the original and the anonymized routed distances was 6.1 km (interquartile range: 2.4–11.8 km). FF 2 showed analogous fidelity after its hub-and-spoke procedure with a MAE of 5.8 km (interquartile range: 3.1–9.4 km), corresponding to a median relative error of approximately 2%

The trip durations, derived from the routing API using masked coordinates, exhibited nearly identical distributions to the original routed durations, with MAEs < 8 min (FF 1) and < 7 min (FF 2).

The global spatial shift for FF 2 and the per-cluster isometries for FF 1 are rigid transformations, hence all intra-cluster pairwise distances, angles, and relative positions are preserved exactly. Overall geographic spread and directional flow patterns remain visually and statistically intact. Aggregated OD-matrices at NUTS-2 (50 km grid) level show Pearson correlations > 0.89 (FF 1) and > 0.97 (FF 2) between original and anonymized locations. [6] The DBSCAN-derived cluster membership and internal density characteristics are unchanged.

Histograms of departure/arrival times-of-day are identical before and after because time-of-day windows are retained exactly. Hence, any downstream scheduling or time-window feasibility analysis experiences zero distortion from anonymization. No artificial peaks, shifts, or smoothing were introduced. Peak-hour identification analyses are therefore unaffected.

Because vehicle and driver pseudonymization is consistent within each forwarder, and because trip ordering, chaining, and multi-day flags are fully preserved, all per-vehicle and per-driver utilization statistics (trips per day, km per operational day, tour length, idle time between trips, etc.) can be computed identically on the anonymized data.

The combination of (i) exactly preserved time windows, (ii) high-fidelity distances, (iii) fully retained vehicle/driver consistency and multi-day indicators means that the dataset constitutes valid, realistic benchmark instances for vehicle routing problems (VRP), pickup-and-delivery problems with time windows (PDPTW), and dynamic dispatching algorithms. The introduced distance noise is smaller than typical real-world variability (from e.g. traffic, weather, road works) and is therefore unlikely to alter conclusions about algorithm robustness or relative performance.

Overall, the anonymization procedures introduce only bounded and fully documented distortions. The dataset therefore supports valid and reproducible benchmarking for vehicle routing, dispatching, and related algorithms. Researchers should report sensitivity to the quantified error margins.

## Limitations

The dataset is limited to the operational regions of only two freight forwarding companies within Germany. Hence, results may not be directly transferable to other geographic regions or countries with different infrastructure, regulations, or logistics practices.

The dataset primarily captures regional haul distribution trip patterns, defined by a typical daily 480 km radius to a home base [7]. Consequently, the data may not be representative of long haul logistics patterns involving extended international routes, very long daily driving distances, or fundamentally different operational models (e.g., dedicated long-distance shuttle services or cross-border multi-drop tours).

No data on cargo weight, volume, commodity type, or loading/unloading activity details are available. This limits direct applicability to studies requiring accurate load factors, freight volume estimation, or payload-dependent energy consumption modeling (electrification studies must incorporate external load assumptions).

Only two freight forwarding companies, with a combined total of only 120 unique trucks, are represented. While the data offer valuable real-world operational depth, they may not capture the full heterogeneity of German road freight operations, including differences in company size, specialization, vehicle type, or regional focus.

Exact original GPS coordinates are not provided. Spatial perturbation introduces a median distance error of around 6 km. This may limit the precision of street-level or curb-side routing replication and very fine-grained spatial analyses, although macro-level origin–destination patterns, corridor flows, and relative spatial relationships remain well preserved. Researchers needing exact coordinates for micro-level validation would require access to the original non-anonymized data under appropriate confidentiality agreements (not publicly available).

The original dataset did not include payload weights, cargo types, or commodity details. Consequently, the dataset cannot be used directly for analyses requiring load factors (e.g., precise energy consumption modelling for battery-electric vehicle feasibility, payload-optimized routing, or freight volume statistics).

The dataset does not include stop-level activity codes, such as “freight drop-off”, “pick-up”, “administrative tasks”, “driver rest break”, or “fueling”, or land-use classification, such as “warehouse”, “factory”, “retail store”, “depot”, etc. Hence, no detailed activity analysis is possible. For FF 2 with its hub-and-spoke model, all trip origins are the company depot (typically outbound-loaded trips or inbound returns), while destinations are customer sites. For FF 1 with its direct haul operations, origins and destinations represent sender/receiver addresses and alternate between pick-up and drop-off functions across chained trips. This can also not be reliably derived post-anonymization due to coordinate perturbation.

Additional limitations are the missing driver information for FF 2, that all vehicles are internal-combustion engine trucks and hence no alternative powertrains are present, that the data reflect 2024 operations only, and that exact vehicle make/model or axle configurations are not recorded in the original dataset.

The possible impacts on downstream applications of these limitations of the anonymized dataset are stated in Table 2.

**Table 2:** Expected limitations introduced by the anonymization procedures for common downstream applications of the dataset.

Dataset application	Impact of Anonymization
vehicle routing / VRP benchmarking	trip distances preserved (median error of 6.1/5.8 km), spatial topology retained, absolute depot/stop coordinates are shifted but internal structure is preserved
dispatching optimization	time windows, vehicle IDs, driver IDs, and day sequences are preserved, no payload data available, capacity constraints cannot be modelled
freight truck flow forecasting	temporal sequence and time-of-day distributions are fully preserved, seasonal/calendar alignment is deliberately obscured
EV electrification / range studies	trip distance distributions are well-preserved (median error of 6.1/5.8 km), no cargo load data available hence limiting energy consumption modelling accuracy
ML algorithm development	rich feature set (location, time, distance, driver, vehicle) retained, ground-truth calendar context unavailable

## Ethics Statement

The authors have read and complied with the ethical guidelines for publication in Data in Brief and confirm that the current work does not involve human subjects, animal experiments, or any data collected from social media platforms. The data were obtained with the explicit permission of the participating freight forwarding companies. All data have been fully anonymized to ensure that no individual persons or specific customer business relationships can be identified. The publication of this dataset does not violate any trade secrets or privacy agreements.

## CRediT Author Statement

**Tom Winkler:** Conceptualization, Methodology, Software, Validation, Formal analysis, Investigation, Data curation, Writing – original draft preparation, Writing – review and editing, Visualization; **Marcel Brödel:** Investigation, Data curation, Writing – review and editing; **Laura Heindl:** Methodology, Software, Validation, Formal analysis, Investigation; **Niclas Klein:** Investigation, Data curation, Writing—review and editing; **Anna Paper:** Investigation, Data curation, Writing—review and editing

## Abbreviations

The following abbreviations are used in this manuscript:

**Table:** 

API	application programming interface
CSV	comma-separated values
DBSCAN	Density-Based Spatial Clustering of Applications with Noise
MAE	median absolute error
NUTS	nomenclature des unités territoriales statistiques
OD	origin-destination
PDPTW	pickup-and-delivery problem with time windows
TMS	Transport Management System
VRP	vehicle routing problem

## Data Availability

ZenodoTUMFTM/heavy-duty-vehicle-order-dataset (Original data).
